# Episcleritis with Degeneration of Iris-Iridectomy for Restoration of Sight

**Published:** 1884-05

**Authors:** Chas. W. Hickman

**Affiliations:** Augusta, Ga.; Professor of Eye and Ear Diseases in the Medical Department of the University of Georgia


### EPISCLERITIS WITH DEGENERATION OF IRIS—IRIDECTOMY FOR RESTORATION OF SIGHT.

BY CHAS. W. HICKMAN, M.D., AUGUSTA, GA.

*Professor of Eye and Ear Diseases in the Medical Department of the University of Georgia.*

Episcleritis is an inflammation involving, at first, the episcleral tissue and soon extending to the sclera itself. The inflammation is characterized by hyperæmia, and afterwards swelling of a circumscribed region, which may be near the margin of the cornea or at any other portion of the sclera. Most generally, by preference, it makes its appearance at or near the insertion of one or more of the recti muscles. This inflammation soon assumes a dark-blue shade, and may be diffused through the general surface of the membrane, or may be conspicuous by forming a bluish-black mound about the size of a buck shot at any one or several parts of the sclerotic.

In quite a good percentage of cases the affection presents a tendency to run its course to a favorable termination in a period varying from four to eight weeks. Such, however, is not always the case. A marked tendency to relapses frequently shows itself. As fast as one tumor seems to yield another springs up, until finally more or less of the whole affected portion of the sclerotic appears thinned and bluish, the intraocular tension greatly increased, and the eyeball presenting, in fact, a distended bluish appearance with one or more of the before-mentioned tumors seen here and there. Then again, should the inflammation be situated near the margin of the cornea, it may press upon the ciliary circulation so as to materially interfere with the nutrition of the cornea, leaving that body subjected to the ills naturally attendant upon a defective nutrition.

Finally, the iris may participate in the inflammation, and should this not be early recognized and dealt with, serious consequences might result, such even as occlusion of the pupil and degeneration of the tissue, as the following case will show:

The patient was a female, aged thirteen, brought to me by her grandmother, with the statement, that for nearly a year she had suffered from an inflammation of her right eye. The attacks seemed to return almost as soon as any improvement was gained, until finally sight was lost. The eye presented a bluish, distended appearance, the intra-ocular tension quite marked and a large bluish prominence not far from the upper and outer edge of the cornea, and with one or two smaller ones scattered at other places. The iris had participated in the inflammation, leaving the pupil occluded. An iridectomy was advised and the patient put under chloroform, but the iris was found so decayed that it was only by tearing away with the forceps a small fragment that an opening of sufficient size could be made in order to give the patient sight.

The two affections most liable to be confounded with episcleritis, are phlyctenular ophthalmia and cyclitis. The former is easily recognized by the fact of its being an ulcerated or herpetic spot on the surface of the conjunctiva, and with a leash of blood-vessels running towards it, while in episcleritis the inflammation is beneath the conjunctiva, and soon assumes the dark blue shade, the injection at the same time being more diffused and extensive. In cyclitis, while we may at times have a slightly bluish look around the ciliary body, yet it is nothing like that which characterizes episcleritis. Besides, in cyclitis, the pain is often so great and the eye so exquisitely sensitive, that the patient shrinks from the mere thought of placing the tip of the finger on it.

Lastly, in cyclitis vision is much impaired, while in episcleritis it is often not disturbed.

